# The epidemiology of non-viral gastroenteritis in New Zealand children from 1997 to 2015: an observational study

**DOI:** 10.1186/s12889-018-6229-4

**Published:** 2019-01-05

**Authors:** Emma Jeffs, Jonathan Williman, Natalie Martin, Cheryl Brunton, Tony Walls

**Affiliations:** 1 Department of Paediatrics, University of Otago, PO Box 4345, Christchurch Mail Centre, Christchurch, 8140 New Zealand; 2 Department of Population Health, University of Otago, PO Box 4345, Christchurch Mail Centre, Christchurch, 8140 New Zealand

**Keywords:** Epidemiology, Bacterial gastroenteritis, Protozoal gastroenteritis, *Campylobacter*, *Shigella*, *Salmonella*, *Yersinia*, *Escherichia coli*, *Giardia*, *Cryptosporidium*

## Abstract

**Background:**

Acute gastroenteritis is a substantial cause of hospitalization in children. *Shigella, Salmonella, Campylobacter, Yersinia,* enterotoxigenic *Escherichia coli* (ETEC)*, Giardia and Cryptosporidium* are gastrointestinal pathogens that are notifiable in New Zealand (NZ). The impact of these infections in the pediatric population has not yet been analyzed. The aim of this study was to describe the epidemiological trends in disease notifications and hospital admissions due to non-viral gastroenteritis in NZ children.

**Methods:**

In this population-based descriptive study, age-specific and age-standardized notification and hospital admission rates were analyzed from 1997-to-2015 for *Shigella, Salmonella, Campylobacter, Yersinia,* ETEC*, Giardia and Cryptosporidium* infections in children < 15 years of age. Variations in disease by gender, age, ethnicity and geography were described.

**Results:**

From 1997-to-2015 there were 74,454 notifications (57.6% male) and 3192 hospitalizations (56.4% male) due to non-viral gastroenteritis in NZ children aged < 15 years. There was an overall trend towards a reduction in disease notifications and hospitalizations, however each disease showed a unique pattern of change over time. *Campylobacter* was the pathogen most frequently notified, accounting for 51.7% of notifications and 43.4% of hospitalizations. The hospitalization-to-notification ratios were, from highest to lowest, *Salmonella typhi* (1:1.09), *Shigella* (1:4.0), ETEC (1:7.81), nontyphoidal *Salmonella* (1:13.1), *Campylobacter* (1:27.8)*, Yersinia* (1:29.2)*, Cryptosporidium* (1,33.4), and G*iardia* (1,72.5). Compared to females, male notification rates were approximately 40% higher for *Campylobacter*, 25% higher for *Giardia* and *Yersinia*, and 15% higher for *Cryptosporidium* and nontyphoidal *Salmonella* (*p* < 0.001). Notification rates were highest in children 1–4 years, with the exceptions of nontyphoidal *Salmonella, Salmonella typhi* and *Yersinia*. Notification rates for nontyphoidal *Salmonella and Yersinia* were highest in children < 1 year, and for *Salmonella typhi* those aged 5–9 years. Children < 1 year were most likely to be hospitalized.

**Conclusions:**

The incidence of non-viral gastroenteritis in NZ children reduced during the 19-year period considered. The burden of disease was highest in the community, with only a small percentage of cases requiring hospitalization. This study provides important insight into the non-viral causes of gastroenteritis in NZ children and how environmental influences and changes in food safety practices may have helped to reduce the burden of these diseases in children.

**Electronic supplementary material:**

The online version of this article (10.1186/s12889-018-6229-4) contains supplementary material, which is available to authorized users.

## Background

Acute gastroenteritis is a common illness and a substantial cause of hospitalization in previously healthy young children. In their first three years of life, almost all children will experience at least one episode of acute gastroenteritis [1]. Most cases are self-resolving and do not require treatment, however, older people and young children are at an increased risk of complications. Morbidity due to gastroenteritis is greatest in children aged younger than five years [2].

A diverse range of pathogens are implicated in childhood gastroenteritis including viruses, bacteria and protozoa. Viral infections, and rotavirus in particular, account for the greatest burden of gastrointestinal disease in young children worldwide [3]. In December 2009, the World Health Organization (WHO) recommended that all infants should be routinely immunized to prevent rotavirus disease [4]. The rotavirus vaccination was introduced into the New Zealand (NZ) national schedule in 2014. This has led to a significant reduction in the incidence of viral gastroenteritis in NZ children [5]. Studies reporting the epidemiology of pediatric gastroenteritis in NZ have focused largely on rotavirus and other viral pathogens [5, 6]. There are no published studies reporting on the epidemiology of non-viral gastroenteritis in NZ children.

The total burden of disease due to foodborne and waterborne gastrointestinal infection in NZ is high. There are an estimated 4.17 to 5.16 million cases of acute gastroenteritis annually, and the incidence of disease in the community is estimated at 1.11 episodes per person per year [7, 8]. NZ has a notifiable disease monitoring system whereby certain communicable diseases for which investigation and public health action are required are notifiable to the local Medical Officer of Health. Diseases may be made notifiable by the Ministry of Health (MoH). This may be to allow prompt public health action, such as the provision of prophylaxis to contacts (eg. Meningococcal disease), to allow for environmental investigation of potential sources (eg. food and water borne diseases) or to monitor the incidence of vaccine preventable diseases (eg, mumps, measles, rubella). Some diseases are also notifiable as part of NZ’s commitment to implementation of the International Health Regulations (eg. Yellow Fever, Middle Eastern Respiratory Syndrome (MERS)).

Data on notifiable diseases are reported to The Institute of Environmental Science and Research (ESR) for disease surveillance and monitoring on behalf of the NZ MOH. ESR is one of ten NZ Crown Research Institutes (CRI) established in 1992, after the reorganisation of the Government-owned Department of Scientific and Industrial Research (DSIR) and science units from the Department of Health. As well as carrying out research, it is also responsible for surveillance and reporting of notifiable diseases in NZ. The list of notifiable disease in NZ can be found at https://www.health.govt.nz/our-work/diseases-and-conditions/notifiable-diseases [9]. Notifiable bacterial pathogens include *Campylobacter, Shigella, Salmonella, Yersinia* and verotoxin-producing or shiga toxin-producing enterotoxigenic *Escherichia coli* (ETEC)*,* and notifiable protozoal pathogens include *Giardia* and *Cryptosporidium. Campylobacter* has been notifiable since 1980, ETEC since 1993, and all other pathogens since at least 1996 (Table 1).

**Table 1:** Year of commencement of ESR notification for diseases in this study, and ICD9-CM codes used to identify hospital admission rates

	Year of commencement of ESR notification	ICD-9-CMA-II codes
*Campylobacteriosis*	1980	A04.5
*Shigellosis* ^*a*^	–	A03
*Salmonellosis (*including *Salmonella typhi* and nontyphoidal *Salmonella)* ^*a*^	–	A02
*Yersiniosis*	1996	A04.6
*Giardiasis*	1996	A07.1
*Cryptosporidiosis*	1996	A07.2
ETEC ^*b*^	1993	A04.0-A04.4

^a^ - It is unclear when reporting for *Shigellosis* and *Salmonellosis* commenced, but ESR has informed the authors of this study that these have likely been notifiable since the Health Act of 1956. Yearly totals are available to 1960

^b^ - ETEC was notifiable from 1993 as *‘food poisoning’*. In 1996, this changed to being notifiable as *‘acute gastroenteritis’*, and then in 2012 became notifiable as *‘*verotoxin-producing or shiga toxin-producing *Escherichia coli’*

Surveillance data for NZ adults suggest the incidence of notifications for bacterial gastroenteritis is decreasing over time; a trend that has been particularly evident since the mid-2000s [10]. The incidence of protozoan illness has remained static over time [10]. *Campylobacter* is the most frequently identified gastrointestinal pathogen, followed by nontyphoidal *Salmonella* and *Giardia* [11]. *Campylobacter* is the most frequently notified gastroenteric disease in NZ and accounts for at least 35% of all notifiable disease reports (158.9 cases per 100,000 in the population in 2016) [10]. *Campylobacter* is also frequently implicated in outbreaks of disease, some of which have been large. For example, a recent outbreak of *Campylobacter* illness caused by contamination of drinking water occurred in Havelock North, NZ in August 2016, during which 5500 of the town’s 14,000 residents were estimated to have become ill with the disease [12].

The NZ health system is predominantly publicly-funded with services provided by public, private and nongovernmental sectors. The country is divided into twenty District Health Boards (DHB's) that are responsible for the planning and funding of health services for their geographical areas. Primary healthcare (including general practitioners) is free for all children aged 13 years and under and is subsidized for residents and citizens. Tertiary-level care (hospitals) is free-of-charge for all and there is no requirement for private healthcare insurance. The health status of the NZ population overall is high, however, there are significant inequalities in the health of Māori, the indigenous people [13].

Acute gastroenteritis affects children in greater numbers than adults [14], however, systematic analysis of the impact of non-viral gastroenteritis in NZ children has not been reported previously. The aim of this study was to determine the burden of disease due to non-viral gastroenteritis in NZ children by describing trends in hospital admissions and disease notifications from 1997 to 2015.

## Methods

### Datasets

In this population-based descriptive study that aimed to describe the epidemiological trends in disease notifications and hospital admissions due to non-viral gastroenteritis in NZ children, extracts from two data sets were analyzed for the period 1 January 1997 to 31 December 2015: The National Minimum Dataset (NMDS) and ESR’s notifiable diseases database, EpiSurv. The NMDS is a national dataset of routinely collected public and private hospital discharge information that includes coded clinical data for inpatients and day patients [15]. The ESR database contains data on notifiable diseases. All cases of notifiable disease resulting in hospital admission included in the NMDS should, theoretically, also be included in ESR’s database.

The NMDS was introduced in 1999. The original NMDS was implemented in 1993 and retrospectively-loaded with public hospital discharge information from 1988 [15]. The NMDS dataset included diseases defined according to their International Classification of Diseases – Clinical Modification 9 (ICD-9-CM) codes (Table 1). ESR commenced standardized online reporting in 1997 and therefore 1997 was elected as the start point for data analysis.

The data extracted from the NMDS database included: primary diagnosis (by ICD-9-CM code), provider agency code, agency name (the name of the admitting hospital), facility code, facility name, gender code, prioritized ethnic code, patient age at discharge, financial year, year of data, short stay data, month of data and number of discharges.

ESR data were obtained from a web-based portal, EpiSurv, which is NZ’s national database for notifiable disease surveillance and is operated by ESR on behalf of the NZ MoH. Under the Health Act 1956, healthcare professionals are required to inform their local Medical Officer of Health on suspicion or diagnosis of a case of any notifiable disease [9]. Medical Officers of Health and local public health unit staff investigate these notifications and complete EpiSurv case report forms online. Laboratory cases are routinely submitted to ESR to be loaded onto EpiSurv.

The data extracted from the ESR database included: EpiSurv number, case status, report date, DHB, age in years, sex, ethnicity (selected and prioritized), onset date information, death details, and clinical details.

Subgroup analysis was performed for Māori children because there are significant health inequalities for this group when compared to non-Māori, including high rates of other infections such as invasive bacterial infections (bacterial meningitis [16] and septicemia [17]), acute rheumatic fever [18], and skin and soft tissue infections [19].

Reported cases of *Salmonella* were subdivided in order to analyze data on nontyphoidal *Salmonella* and *Salmonella typhi* separately*.* Our study included non-viral causes of gastroenteritis which are notifiable under the Health Act in NZ. While cholera is a notifiable disease, it is not endemic in New Zealand, and the very occasional cases seen are in travellers from endemic countries. Helminth infestations were also not included in the study as only cestode infestations (cystercicosis, taeniasis and hydatids) are notifiable and these were uncommon in the study period.

### Statistical analysis

Data were analyzed using the statistical software ‘R’ *(R version 3.3.2 (2016-10-31))*. Notifications and hospitalizations were grouped by disease, year, age (categorized as < 1, 1 to 4, 5 to 9, and 10 to 14 years), gender (female, male), and ethnicity (Māori, non-Māori) both separately and in combinations. Where ethnicity was unknown, cases were included in total counts but excluded from ethnic comparisons. Group specific incidence rates were calculated using the estimated resident population at 30 June (derived from NZ Census data from Statistics NZ) as the denominators. Standard methods were used to directly age-standardize incidence rates to the WHO 2000–2025 population [20].

Changes in log age-standardized incidence rates of disease over time were analyzed using joinpoint regression models [21], fitted using the Joinpoint Regression Program (version 4.5.0.1) [22]. Models were allowed up to four joinpoints, with each joinpoint being no less than two years from another joinpoint or the ends of the data. These models were used to calculate the average annual percentage change (AAPC) in age-standardized incidence rates for the full data range of each disease, as well as selected time periods. The models were also used to assess differences in trends over time between selected subgroups. Comparisons in incidence rates by age, gender, and ethnicity were also investigated by using generalized additive poisson regression models to calculate incidence rate ratios with 95% confidence intervals. Year of observation was adjusted for in models by fitting it as a penalized thin plate regression spline [23].

## Results

### Overall summary

In the 19-year period from 1997 to 2015 there were 74,454 notifications (57.6% male) and 3192 hospitalizations (56.4% male) due to non-viral gastroenteritis in NZ children. *Campylobacter* was the disease most frequently notified and accounted for 51.7% of all notifications and 43.4% of all hospitalizations. Notification rates were highest in children aged 1–4 years of age.

### Notifications versus hospitalizations

**Fig. 1:**
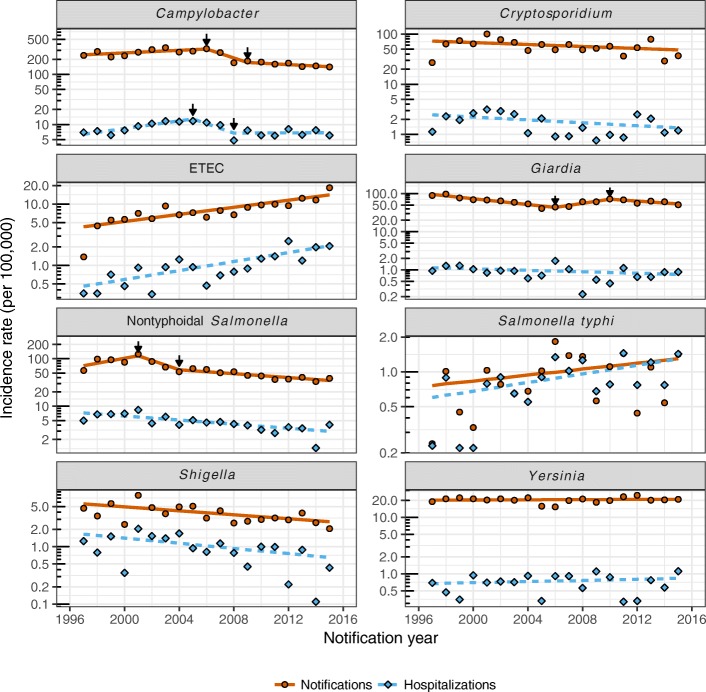
Notifications and hospitalizations by disease and year. Points show annual age-standardized incidence rates. Lines represent joinpoint predicted trend lines with black arrows demarking changes in the slope of trend lines

Hospitalization rates were typically much lower than the corresponding notification rates for each disease (Fig. 1). The hospitalization to notification ratios were, in order from highest to lowest, *Salmonella typhi* (1:1.09), *Shigella* (1:4.0), ETEC (1:7.81), nontyphoidal *Salmonella* (1:13.1), *Campylobacter* (1:27.8)*, Yersinia* (1:29.2)*, Cryptosporidium* (1:33.4), and G*iardia* (1:72.5).

### Time trends

Whilst there was an overall trend towards a reduction in both notifications and hospitalizations, particularly since the mid-2000s, each disease showed a unique pattern of change over time (Fig. 1, Table 2). Notification and hospitalization rates have been decreasing consistently for *Shigella* since at least 1997, and for nontyphoidal *Salmonella* since 2001 (AAPC notification since 2001 = − 8.20, 95%CI -15.08 to − 0.77, *p* = 0.031). There was weak evidence that rates were also decreasing for *Cryptosporidium,* whilst rates for *Yersinia* have remained quite stable*.* ETEC notifications and hospitalizations increased substantially over the study period, and *Salmonella typhi* hospitalization rates also increased by, on average, 4.4% per year.

**Table 2:**
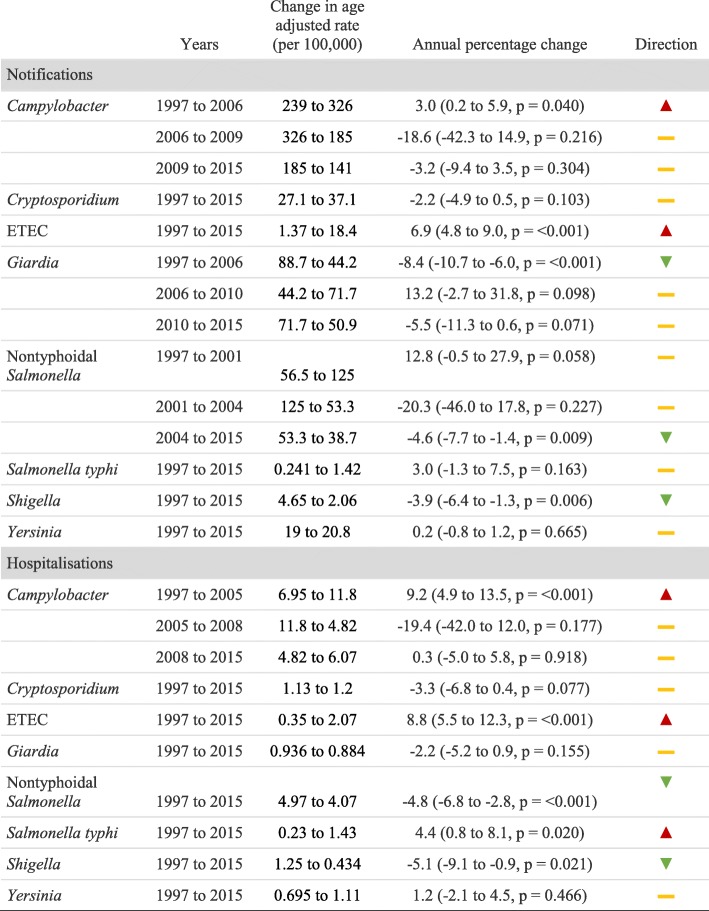
Changes in notification and hospitalization incidence rates over time

There was a noticeable increase in *Campylobacter* notifications and hospitalizations at the beginning of the study period with age-adjusted notification rates peaking at 326 per 100,000 in 2006. Notification rates almost halved to around 171 per 100,000 in 2008 and have gradually continued to decrease to a low of 141 per 100,000 in 2015. However, *Campylobacter* hospitalization rates have remained around 6.6 per 100,000 since 2008. Age-adjusted notification rates for *Giardia* halved between 1997 and 2006 (from 88.7 to 44.2 per 100,000), but increased to 71.7 per 100,000 in 2010 before resuming on the downward trend. Interestingly, *Giardia* hospitalization rates did not appear to follow this pattern.

### Comparisons by age

Notification rates were highest in children aged 1–4 years of age, with the exceptions of nontyphoidal *Salmonella, Salmonella typhi* and *Yersinia*. Notification rates for nontyphoidal *Salmonella and Yersinia* were highest in infants aged under 1 year, and for *Salmonella typhi* in those aged 5–9 years (Table 3). Whilst notifications for C*ampylobacter* were more frequent in the 1–4 year age group, those under 1 year were most likely to be hospitalized. For all other diseases, differences by age in hospitalization rates were broadly similar to differences in notifications.

**Table 3:** Differences in notification (N) and hospitalization (H) rates by age group as compared to those aged 10–14 years. Values represent incidence rate ratios (95% confidence intervals) adjusted by year and gender

Disease	Source	Age, < 1 years	Age, 1 to 4 years	Age, 5 to 9 years
*Campylobacter*	N	2.53 (2.43, 2.63)	*3.09 (3.01, 3.18)*	1.18 (1.14, 1.21)
	H	*2.25 (1.89, 2.68)*	1.52 (1.33, 1.73)	0.76 (0.65, 0.88)
*Cryptosporidium*	N	2.53 (2.28, 2.81)	*7.03 (6.58, 7.51)*	2.11 (1.96, 2.27)
	H	2.85 (1.65, 4.92)	*5.38 (3.74, 7.74)*	2.18 (1.47, 3.23)
ETEC	N	8.33 (6.50, 10.7)	*11.0 (8.9, 13.5)*	2.17 (1.71, 2.75)
	H	5.56 (2.94, 10.5)	*7.20 (4.37, 11.9)*	1.85 (1.04, 3.28)
*Giardia*	N	4.12 (3.71, 4.57)	*10.6 (9.82, 11.4)*	3.25 (3.00, 3.52)
	H	5.34 (2.85, 10.0)	*5.35 (3.24, 8.82)*	1.43 (0.79, 2.57)
Nontyphoidal *Salmonella*	N	*6.52 (6.06, 7.02)*	5.31 (4.99, 5.65)	1.63 (1.52, 1.75)
	H	*10.8 (8.48, 13.7)*	4.23 (3.38, 5.29)	1.44 (1.11, 1.86)
*Salmonella typhi*	N	0.14 (0.02, 1.02)	1.65 (1.07, 2.55)	*1.89 (1.26, 2.83)*
	H	0.25 (0.06, 1.01)	1.26 (0.82, 1.94)	*1.48 (1.00, 2.20)*
*Shigella*	N	1.74 (1.14, 2.65)	*4.57 (3.59, 5.81)*	2.52 (1.96, 3.24)
	H	2.24 (0.92, 5.44)	*6.02 (3.51, 10.3)*	3.98 (2.30, 6.88)
*Yersinia*	N	*9.30 (8.21, 10.5)*	6.27 (5.61, 7.00)	1.06 (0.92, 1.22)
	H	*19.4 (10.3, 36.6)*	3.79 (1.97, 7.29)	1.93 (0.96, 3.88)

### Comparison by gender

Compared to females, male notification rates were approximately 40% higher for *Campylobacter*, 25% higher for *Giardia* and *Yersinia*, and 15% higher for *Cryptosporidium* and nontyphoidal *Salmonella* (*p* < 0.001 in all cases, Table 4). There was no evidence for a difference in notification rates for ETEC, *Salmonella typhi*, or *Shigella* (*p* > 0.8 in all cases). Differences in hospitalization rates were only observed potentially due to the relatively low numbers of cases for the other diseases.

**Table 4:** Differences in notification and hospitalization rates by gender and ethnicity. Values represent incidence rate ratios (95% confidence intervals) adjusted by year and age

	Male vs. Female		Māori vs. non-Māori	
	Notifications	Hospitalizations	Notifications	Hospitalizations
*Campylobacter*	1.43 (1.35, 1.51)	1.51 (1.35, 1.69)	0.27 (0.25, 0.30)	0.60 (0.52, 0.70)
*Cryptosporidium*	1.16 (1.04, 1.30)	0.91 (0.73, 1.14)	0.34 (0.28, 0.41)	0.74 (0.54, 1.01)
ETEC	1.00 (0.90, 1.12)	0.88 (0.67, 1.15)	0.43 (0.36, 0.51)	0.98 (0.68, 1.41)
*Giardia*	1.25 (1.17, 1.33)	1.08 (0.83, 1.40)	0.31 (0.27, 0.34)	1.13 (0.82, 1.56)
Nontyphoidal *Salmonella*	1.11 (1.02, 1.20)	1.03 (0.88, 1.20)	0.37 (0.32, 0.42)	0.72 (0.59, 0.89)
*Salmonella typhi*	1.01 (0.74, 1.39)	1.03 (0.77, 1.37)	0.72 (0.27, 1.94)	1.24 (0.20, 7.55)
*Shigella*	1.04 (0.82, 1.31)	0.80 (0.61, 1.05)	0.80 (0.56, 1.12)	1.41 (0.95, 2.09)
*Yersinia*	1.25 (1.15, 1.35)	0.95 (0.76, 1.19)	0.34 (0.30, 0.39)	1.15 (0.83, 1.60)

### Comparisons by Māori

Approximately 20% of cases were missing ethnicity data in EpiSurv records prior to 2009, but the proportion of missing ethnicity data subsequently decreased markedly to around 3.5%. For all the diseases except *Salmonella typhi* and *Shigella*, notification rates in Māori children were approximately 60 to 70% lower than in non-Māori children (Table 4). Ethnic differences in hospitalization rates were only evident for *Campylobacter* (*p* < 0.001) and nontyphoidal *Salmonella* (*p* = 0.003), and suggestive for *Cryptosporidium* (*p* = 0.066). *Shigella* was the only disease that suggested higher hospitalization rates in Māori (*p* = 0.092).

## Discussion

This is the first study in NZ to analyze longitudinal data on childhood gastroenteritis caused by non-viral pathogens. The trend towards a decline in both notifications and hospitalizations for most non-viral pathogens included in this study is reassuring. Most of these pathogens are predominantly foodborne, and the observed decline may have been contributed to by changes in food safety, such as food industry standards relating to food handling and general hygiene, as well as increased consumer awareness of food safety [24]. The decline may also have been contributed to by changes in the food production sector, including farming, such as increased mechanization and more stringent monitoring of food safety indicators.

Consistent with several other international studies [25–27], *Campylobacter* was identified as the pathogen associated with the majority of notifications and hospitalizations. Hospitalization rates increased and decreased in parallel with notification rates, thus it is unlikely these results are artefactual. Our results are consistent with trends observed in the adult population in NZ, in which notification rates for *Campylobacter* peaked in 2006 (> 380 per 100,000) and were lowest in 2008 (161.5 per 100,000) [10].

Between 2004 and 2008, and most notably between 2006 and 2008, there was a reduction in *Campylobacter* notifications that was consistent across all age groups. This reduction coincided with the introduction of a range of voluntary and regulatory interventions to reduce *Campylobacter* contamination in poultry [28]. In late 2006, the NZ Food Safety Authority (formerly part of the Ministry of Agriculture and Fisheries - now the Ministry for Primary Industries) released guidance aimed at reducing the incidence of poultry-associated foodborne *Campylobacter* disease. This was in response to research that indicated poultry contributed disproportionately to the incidence of *Campylobacter* disease in NZ [28, 29]. Interventions included the development and implementation of microbiological surveillance activities and increased reporting. The observed reduction in *Campylobacter* incidence in children seen in this study provides evidence of the positive effects of a food safety measure on child health. Whilst these findings are encouraging, there is some suggestion from our analysis that *Campylobacter* notification rates may have plateaued since 2008. This may, in part, be attributable to recent outbreaks, which have been water, rather than food-borne, or other changes such as the increasing popularity of the consumption of raw milk. This observation warrants purposeful surveillance.

Nontyphoidal *salmonellae* are also common bacterial pathogens associated with acute gastroenteritis in children [26, 30] and the leading cause of childhood bacterial enterocolitis requiring hospitalisation [31]. Although the food safety interventions mentioned above were targeted predominantly at *Campylobacter*, they may also have served to reduce the incidence of disease due to nontyphoidal *Salmonella,* which is also commonly transmitted via poultry. The observed reduction in the incidence of nontyphoidal *Salmonella* between 1997 and 2016 (as for *Campylobacter*) supports this hypothesis.

Unlike for nontyphoidal *Salmonella*, the rates of notification and hospitalization for *Salmonella typhi* were very similar. This finding is not surprising given that infection with *Salmonella typhi* is more likely to present with severe disease requiring hospitalization.

The number of notifications of ETEC infection increased steadily after 1997, reaching a peak of 16 cases per 100,000 in the population across all age groups in 2016. This increase may, in part, be due to changes in laboratory testing practices over that period, with increasingly sensitive assays and algorithms used for the detection of *Escherichia coli (E. coli)* [32] It could also have been contributed to by improvements in surveillance due to increased public health concern [32]. However, the increased rates of notification and hospitalization in the under one and 1–4 year age groups compared to those > 5 years are notable. Jaros et al. (2014) reported that the highest rate of *E. coli* infections in their national prospective case-control study of confirmed cases of Shiga toxin-producing *E. coli* in NZ occurred in the 1–4 year age group. The risk to children was increased further if there was a family member in contact with animals other than household pets. That study provides some support for the contention that the higher rates of disease in babies and children under 5 years of age found in our study is unlikely to be accounted for by changes in laboratory testing practices in those ≤4 years of age.

Exposures to farming environments have been reported as risk factors of sporadic ETEC infections, particularly in young children [33, 34]. Sheep and, in particular, cattle, are considered an important reservoir for ETEC and are the primary source of foodborne and environmental outbreaks in humans [32]. Our study observed higher rates of ETEC infections in DHB regions with more intensive dairy farming compared to those DHB regions with less intensive dairy farming (Additional file 1: Figure S1).

Māori children had lower notification rates for disease when compared to non-Māori children. This is an unexpected finding. A previous study has reported that risk for morbidity due to infectious disease is higher in Māori compared to non-Māori, and also that infectious disease incidence in Māori has risen considerably over time, resulting in pronounced gradients of disease inequality [35]. However, a lower incidence of *Campylobacter* in Māori adults has been reported previously [8, 36]. Despite Māori people being more likely to live in farming regions [37], their incidence of notified *Campylobacter* disease does not correspond with the higher rates seen overall in these areas. Disparities in Māori access to health care are well documented and may be contributing to the lower rates of notification in this population [38]. In order for notification to occur, children must present to a health care provider and be tested. Hence, the results observed could be due to lower rates of both presentation and subsequent testing.

### Strengths and limitations

NZ has a robust disease notification system regulated under the Health Act 1956. Notification can be initiated on the basis of ‘clinical suspicion’ by a medical practitioner, but is usually based on the isolation of a pathogen from a clinical sample. All admissions to public hospitals are recorded and data on hospital admissions collated and held nationally. This study has used data from the two most comprehensive national datasets in our analysis.

It is widely accepted that the incidence of gastroenteritis in NZ is underreported. Lake et al. (2010) reported that for every notified case of acute gastroenteritis in NZ, there were an estimated 222 cases in the community that were not reported [7]. It is estimated that 0.4% of community cases of acute gastroenteritis are notified to the national surveillance system [39]. Recent changes to reporting methods, alongside improvements to ETEC testing and detection, raise the possibility that there could have been more systematic underreporting and subsequent underestimation of disease earlier on in the study period.

Although considerable evidence has been published suggesting that interventions implemented by the food industry may have contributed to the decline in the incidence of *Campylobacter* gastroenteritis and disease due to nontyphoidal *Salmonella* in NZ, several alternative explanations should be considered. These include the possibility of surveillance artifact, a decline in the relative proportion of food versus waterborne disease, and changes in consumer behavior, such as increased awareness of food hygiene.

Our observations in Māori children regarding the low rates of notification are concerning and warrant further investigation. Of note, almost one fifth of cases were missing ethnicity data in ESR EpiSurv records prior to 2009, although the proportion of cases with missing ethnicity data decreased markedly after that. As such, differences in rates by ethnicity prior to 2009 should be interpreted with caution. Misclassification of ethnicity is also of concern and may have contributed to underestimation of disease incidence in Māori children.

## Conclusions

The burden of disease due to non-viral gastroenteritis in NZ children is highest in the community setting. The overall incidence of these infections is reducing over time with only a small proportion of cases needing hospital admission. Disparities in access to healthcare may have led to Māori children having lower rates of notification for non-viral gastroenteritis, and this warrants further investigation. This study provides an important insight into the non-viral causes of gastroenteritis in NZ and how changes in food safety practices may have helped to reduce the burden of these diseases in children. It also highlights the potential importance of other environmental influences on the incidence of some of the most serious of these infections, disease due to ETEC*.*

## Additional file

Additional file 1: **Figure S1.** Notification rates for ETEC by District Health Board. Points represent observed crude incidence. Lines and shading represent incidence with 95% confidence intervals predicted by Poisson regression models assuming a constant annual percentage change (APC). The APC is displayed in text with 95% confidence interval. Key: Areas with intensive dairy farming. Percentages represent the regional distribution of dairy cows (2016/17): North Island of NZ: Waikato (23.0%), Taranaki (9.7%), Northland (5.5%). South Island of NZ: Canterbury (13.8%), Southern (11.6%), Otago (5.3%), South Canterbury (5.0%). * All others < 5%. (EPS 61 kb)

## Abbreviations

AAPC: Average annual percentage change
DHB: District Health Board
ESR: The Institute of Environmental Science and Research Ltd.
ETEC: Verotoxin-producing or shiga toxin-producing enterotoxigenic *Escherichia coli*
ICD-9-CM codes: International Classification of Diseases – Clinical Modification 9
MoH: Ministry of Health, New Zealand
NMDS: National Minimum Dataset
NZ: New Zealand
WHO: World Health Organization
